# The nested hierarchy of self and its trauma: In search for a synchronic dynamic and topographical re-organization

**DOI:** 10.3389/fnhum.2022.980353

**Published:** 2022-09-02

**Authors:** Andrea Scalabrini, Clara Mucci, Georg Northoff

**Affiliations:** ^1^Department of Human and Social Sciences, University of Bergamo, Bergamo, Italy; ^2^Faculty of Medicine, Centre for Neural Dynamics, The Royal's Institute of Mental Health Research, Brain and Mind Research Institute, University of Ottawa, Ottawa, ON, Canada; ^3^Mental Health Centre, Zhejiang University School of Medicine, Hangzhou, China; ^4^Centre for Cognition and Brain Disorders, Hangzhou Normal University, Hangzhou, China

**Keywords:** self, synchrony, nestedness, trauma, dissociation, now moments

## Abstract

The sense of self has always been a topic of high interest in both psychoanalysis and most recently in neuroscience. Nowadays, there is an agreement in psychoanalysis that the self emerges from the relationship with the other (e.g., the caregiver) in terms of his/her capacity to attune, regulate, and synchronize with the emergent self of the infant. The outcome of this relational/intersubjective synchronization is the development of the sense of self and its regulatory processes both in dynamic psychology and neuroscience. In this work, we propose that synchrony is a fundamental biobehavioral factor in these dialectical processes between self and others which shapes the brain–body–mind system of the individuals, including their sense of self. Recently in neuroscience, it has been proposed by the research group around Northoff that the self is constituted by a brain-based nested hierarchical three-layer structure, including interoceptive, proprio-exteroceptive, and mental layers of self. This may be disrupted, though, when traumatic experiences occur. Following the three levels of trauma theorized by Mucci, we here suggest how different levels of traumatic experiences might have an enduring effect in yielding a trauma-based topographic and dynamic re-organization of the nested model of self featured by dissociation. In conclusion, we propose that different levels and degrees of traumatic experience are related to corresponding disruptions in the topography and dynamic of the brain-based three-layer hierarchical structure of the self.

## Introduction

*The self then is the sum of everything we are now, and everything we once were, as well as everything we could potentially become*

*CG Jung, Mysterium Coniunctionis, page 108*.

The sense of self has always been a topic of interest in both psychoanalysis and more recently in neuroscience.

One of the first psychoanalysts who referred to the concept of self was Carl Gustav Jung, who considered the self as a structure that constitutes the whole of the individual, i.e., more than the sum of its constituents. This gives the self as a whole a conceptually superordinate position in relation to the parts, as it operates autonomously as an overarching organizing principle providing the functional unity of mind and body in a constant flux of states of the organism (refer to Jung, “The dynamic and structure of the psyche,” Volume 8, 2014).

Influenced by Kleinian thought, Fordham suggested how the self is based on Freud's structural theory. Although “*not explicitly defined... [it] seems to indicate a concept of wholeness which embraces the ego, super-ego, and id, and is even perhaps something more as well”* (Fordham, 1957, p. 198); that is, it implies a wholeness that unites the structural parts. Accordingly, Strachey (1961, pp. 7–8) and Kernberg (1984, pp. 227–8) also noted that Freud himself never dissociated the Ego from the experiencing self; indeed, Freud preserved the German Ich –Ego– as a mental structure and psychic agency, but also as the personal, subjective, experiential self in all his writing. However, differently from the topography proposed by Freud, Jung describes two different complexes that serve the purpose of an interface, on the one hand with the external world, that is the *persona*, and, on the other hand, of an interface with the inner world, that is the *shadow*, to further emphasize the relational feature embedded in its topography of self. In Junghian terms, *persona* is defined as a social relational interface used to underline the prevalence of the world and the others in its constitution; similarly, the shadow can be considered as an interface between the conscious Ego and the inner world. Finally, the self can be considered the center of the psyche to which the other parts are connected and subordinate. Recently, in an interdisciplinary attempt encompassing neuroscience, psychology, philosophy, and anthropology, it has been hypothesized to consider Ego-I-Self as a continuum that reflects different development stages of the sense of individuality, which emerges from the relationship with the external world (Facco et al., 2019). In this hypothesis, the Ego is considered as the primary component appearing in infancy – related to the psychanalytic concept of primary narcissism. The I represents the evolution of the Ego in relation with the principle of reality. The Self includes both the Ego and I and transcends them according with Jung's individuation process. Although currently, the relational psychodynamic theory has a central role in contemporary psychoanalysis, this perspective was not always recognized. One of the ground-breaking theorists who emphasized the relational component of the development of the self (vs. the intrapsychic conservative component) was Winnicott (1965), who described how the sense of self of the infant can be seen as a result from the “*internalization*” of the empathic and mirroring relationship with the caregiver.

Similarly, Kohut (1971), who is considered the father of self-psychology, conceptualized how a failure in the development of a cohesive sense of the self, depending on the interaction with the environment, leads to a fragmentation of the body, self, mind, and the self-object. Kohut already pointed out the relevance of the animate environment as fundamental for the development of the sense of self. Indeed, contemporary psychodynamic authors, departing from the background of the attachment theory (Schore, 2000, 2001a,b, 2012; Lyons-Ruth, 2003, 2008; Fonagy et al., 2007; Mucci, 2013, 2018a; Beebe and Lachmann, 2014), proposed that the parent–infant dyad can be considered as the first intersubjective encounter that predisposes the development of the self and emphasized how the dual caregiver–infant exchange continuously modulates the formation of the growing subject, organizing the mind-body-brain interoceptive and exteroceptive connections in relation to the other and the world.

Today, there is an agreement in psychoanalysis that the self comes from the other in terms of his/her capacity to attune, regulate, and synchronize to the emergent self of the infant. The outcome of this relational/intersubjective alignment-attunement-synchronization is the development of the sense of self and its regulatory processes.

Contemporarily, there is a renewed discussion on the relationship between psychoanalysis and neuroscience departing from the “ Project for a scientific psychology” (Freud, 1895) reprised recently by Solms in his “New project for a scientific psychology” (Solms, 2020, 2021) and other scholars whom have applied the concept of free energy and predictive coding (Seth and Friston, 2016; Solms and Friston, 2018; Cieri and Esposito, 2019) to explore the mind–brain relationship and the intrinsic relationship between psychoanalysis, the sense of self and its temporal features (refer to Spagnolo and Northoff, 2021; Cieri, 2022).

**Figure 1:**
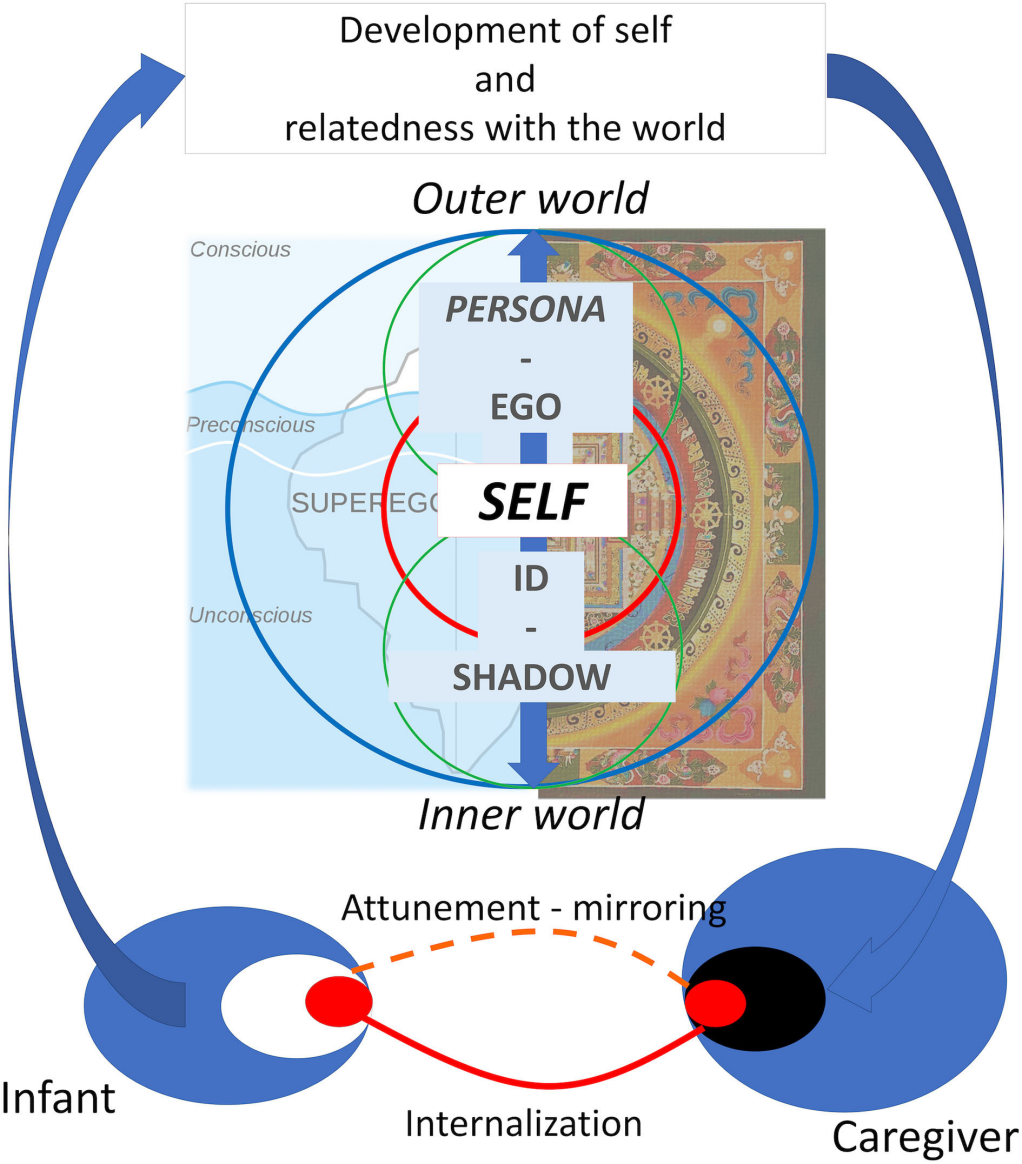
Integrative conception of self in Jung, Freud, and contemporary relational psychoanalysis. Freud's view is represented on the left while Jung's view is on the right of the figure. The bottom of the figure represents the process of self-development through the continuous relational exchange with the caregiver as theorized by contemporary relational psychoanalysis.

Our proposal, similar to Pauli and Jung that considered the material and the subjective worlds as two complementary manifestations of reality (refer to Atmanspacher, 2012; Alcaro et al., 2017), aims for searching the “common currency” between the psyche and the brain as shaped by one's relation with the world (Scalabrini et al., 2020b,c, 2021c; Northoff and Smith, 2022). Here, we propose that synchrony, at a psychological and neuronal level, might be a biobehavioral fundamental factor in the dialectical processes between self and other that shapes the brain–body–mind system of the individual (see Figure 1 for an overview).

## The development of self and relatedness through synchrony

Experience of synchrony finds its priors in early relational experiences with primary caregivers in the context of attachment. In 1975, Colwyn Trevarthen proposed his theory about the origin of human intersubjectivity. He posited that the nonverbal communication between mother and infant, *via* the different senses, serves as an intersubjective emotional communication that, through synchronization, regulates the emotional states of both members of the dyad (Trevarthen, 1993). This synchronized protoconversation co-creates an intersubjective reciprocal system of nonverbal communication where “*the emotions constitute a time-space field of intrinsic brain states of mental and behavioral vitality that are signaled for communication to other subjects and that are open to immediate influence from the signals of these others*” (Trevarthen, 1993, p. 155)

Indeed, biobehavioral synchrony is an important aspect of mother–infant attachment (Feldman, 2007), which contributes to the formation of the sense of self and relatedness (Schore, 2005; Mucci, 2018a,b). Through synchronization, the mother regulates the infant's temperature (Levin, 2006), heart rate (Feldman et al., 2011), sleep, and arousal (Feldman et al., 2002). Mothers regulate their infants' immune function by breastfeeding, synchronizing their gut microbiota and antigen-specific antibodies (Arrieta et al., 2014). Mothers regulate infants' arousal with their voice (by singing, or speaking loudly or softly) (Hofer, 1994; Nakata and Trehub, 2004).

Experience of synchrony can be described as the spatiotemporal coordination between the parent's and the child's nonverbal behavior and communicative signals during social interactions in ways that enhance positive reciprocity and mutual engagement (Stern, 1985; Trevarthen and Aitken, 2001).

Synchrony can thus be seen as a core mechanism underpinning the development of self and relatedness.

Intriguingly, it has been showed how newborns can discriminate between visuo-tactile synchrony and asynchrony (Filippetti et al., 2013). In this experiment, the infant responded to synchronous or asynchronous tactile stimuli caused by another individual, possibly causing the experience of awareness of his/her own body in the infant. This suggests that the infant might start to rudimentally distinguish between self and others through the experience of synchronization vs. asynchronization. This process has also been called “mentalization of the body” (Fotopoulou and Tsakiris, 2017) where the embodied interaction with other people allows the “mentalization” of visceral sensation as experienced subjective feelings.

In this regard, a recent natural and ecological study (Ulmer Yaniv et al., 2021) longitudinally tracked how initial mother–child contact increases mother–child synchrony from infancy across the development until young adulthood. The authors investigated how synchronic experiences enhance the brain's capacity to empathize with other's distinct emotions, particularly in the areas of the brain that have been linked with parent–child synchrony in the parental brain, e.g., the insula (Abraham et al., 2016) and the amygdala (Atzil et al., 2011). Data concerning premature neonates showed that the proximity to the mother's body during incubation is linked with enriched synchronous infant-caregiving experiences reverberating throughout the course of child development. The researchers suggested that synchrony is a natural mechanism by which the human brain connects to others and the social world. In this context “*attachment begins before any sense of self and before any sense of object to attach to*” (Brockman, 2002, p. 90). Indeed, the mother–infant attachment relationship is considered a prior to the development of social synchrony, constituting a dynamic repertoire that can be considered as the capacity to automatically integrate others' emotions together with the abilities to use interoceptive signals to detect others' specific effect as distinguished from one's own effect (self-other distinction).

It has also been shown how the synchronization capacity is affected by the initial opportunities of physical contact in the context of mother–infant attachment. Consistent with the dynamics system theory, preterm infants lacking opportunities of a full maternal physical contact consistently showed lower levels of synchrony compared to infants that did receive contact (Feldman, 2015). Finally, to investigate empathic abilities, the same subjects at the age of 20 performed an fMRI empathy task that involved affective-empathic network constituted prominently by ventromedial prefrontal cortex, temporal pole, insula, and amygdala. The involvement of these regions corresponded to the degree of emotional intensity and the enhanced affective-specific neuronal response in amygdala and insula was related to the synchrony experienced from infancy to adulthood. Focusing now on mothers, it has also been showed that maternal insula and amygdala responses to affective stimuli related to an infant (e.g., infant's crying and laughing) also predict mother–infant synchrony, oxytocin receptors, and play a causal role in the initiation of mammalian mothering. On the other hand, when there is a lesion of the amygdala, maternal caregiving is disrupted (Riem et al., 2011; Lonstein et al., 2015).

Similarly, the human parents' insular response to their infant impacts the child's later capacity to regulate distinct emotions, both positive emotions and distressful ones, in preschool (Abraham et al., 2016). Given that the link between the parental insula and the child's regulation of distinct emotions was again mediated by parent–infant synchrony, it seems that regions of the brain, such as the amygdala and the insula, may provide an integrative foundation for the cross-generational transmission of parenting as mediated by synchronous caregiving.

These findings relate to biobehavioral synchrony, *via* the consistency of caregiving over time, which seems to enhance the development of the child's sense of self and relatedness.

These first synchronous experiences, facilitated through the insula and amygdala as a part of the affective-limbic system, seem to shape the development of a physiological and psychological “baseline” (refer to Northoff, 2016; Scalabrini et al., 2021c, 2022; Northoff et al., 2022) that later assemble predictive models that facilitate the development of a social brain and the self (Apps and Tsakiris, 2014; Atzil et al., 2018) (see also Ebisch et al., 2022 for the role of te insula and empathic traits). The baseline means that the self serves as a reference or standard for any subsequent input processing; more importantly, the self as the baseline serves as a reference for subsequent cognition, affect, and other functions – this is well expressed in the concept of the “psychological baseline” (Northoff and Bermpohl, 2004; Scalabrini et al., 2021c, 2022) and the baseline model of self-specificity (BMSS; Northoff, 2016; Northoff et al., 2022; Scalabrini et al., 2022).

The role of the self as the baseline for any subsequent psychological function is constituted by the above-described dialectical process through synchronization as “relational alignment” (Schore, 2012; Scalabrini et al., 2018; Mucci, 2021a,b) that facilitates the development of the integration of interoceptive signals and exteroceptive information. This is essential for the child's homeostasis and for the development of a social-cognitive-affective-behavioral repertoire related to the sense of self (Craig, 2003; Fotopoulou and Tsakiris, 2017; Atzil et al., 2018).

The quality and the organization of this psycho-physiological baseline needs to be considered as dependent on the relational experiences, first with the attachment figure (e.g., the mother) and, second, with an extended social world which can impact the self both in a positive and in an adverse/traumatic way (refer to Mucci and Scalabrini, 2021). Intriguingly, such internalization of subjective and intersubjective experiences can be rooted in the spontaneous spatiotemporal neuronal activity “baseline” of the brain (Northoff et al., 2020a,b, 2022; Northoff and Scalabrini, 2021). Such baseline on the neuronal level of the brain may well be related to the above-described baseline on the more psychological level, the psycho-physiological baseline: we propose that both baselines are linked and connected through their shared spatiotemporal features, e.g., topography and dynamic as their “common currency” (Northoff et al., 2020a,b).

## Neuronal synchronization: Insula at the crossroads between brain–body–mind

Synchronization at a neuronal level plays an essential role in building the complex spatiotemporal structure and dynamics of the brain. Neuronal synchronization allows integrating neuronal activity from different brain regions (and their respective psychological functions and contents) over long stretches of time and distant regions/networks. Since the correlation between the time series entails synchronization between different regions' neuronal activity, one might suppose that synchronization plays a pivotal role also at a psychological–phenomenological level.

For instance, the group around Tallon-Baudry demonstrated how our psychological sense of self is based on body–brain coupling by temporal synchronization of interoceptive stimuli from the heart and stomach with the brain's spontaneous activity in the insula and other regions, such as the anterior midline regions and visual cortex (Park and Tallon-Baudry, 2014; Park et al., 2014). These findings show that the brain and the temporal structure of its neural activity align or synchronize themselves to the ongoing temporal structure of the body and its ongoing visceral activity in the stomach and heart (Northoff and Huang, 2017; Northoff, 2018). Such “temporo-spatial alignment” (Northoff and Huang, 2017; Northoff, 2018) of the brain to the body, e.g., neuro-visceral monitoring (Tallon-Baudry et al., 2018), is central for constituting the self (first-person perspective). Thus, our sense of self, e.g., our subjective first-person perspective, is based on how our brain is aligned to and thus synchronized with the body, and ultimately the environment or world (Northoff, 2018). Intriguingly, it has been shown at a psychological level that the higher the degree of perception of synchronicity in time and closeness in space toward another person (the animate target) vs. an object (the inanimate target) the higher the degree of self-relatedness (Scalabrini et al., 2019). Similarly, studies conducted by the group around Ansermett and Magistretti investigated the subjective sensation of synchrony or connectedness during a joint task suggesting that the more similar the movements were, the sooner the sensation of synchrony appeared (Llobera et al., 2016).

**Figure 2:**
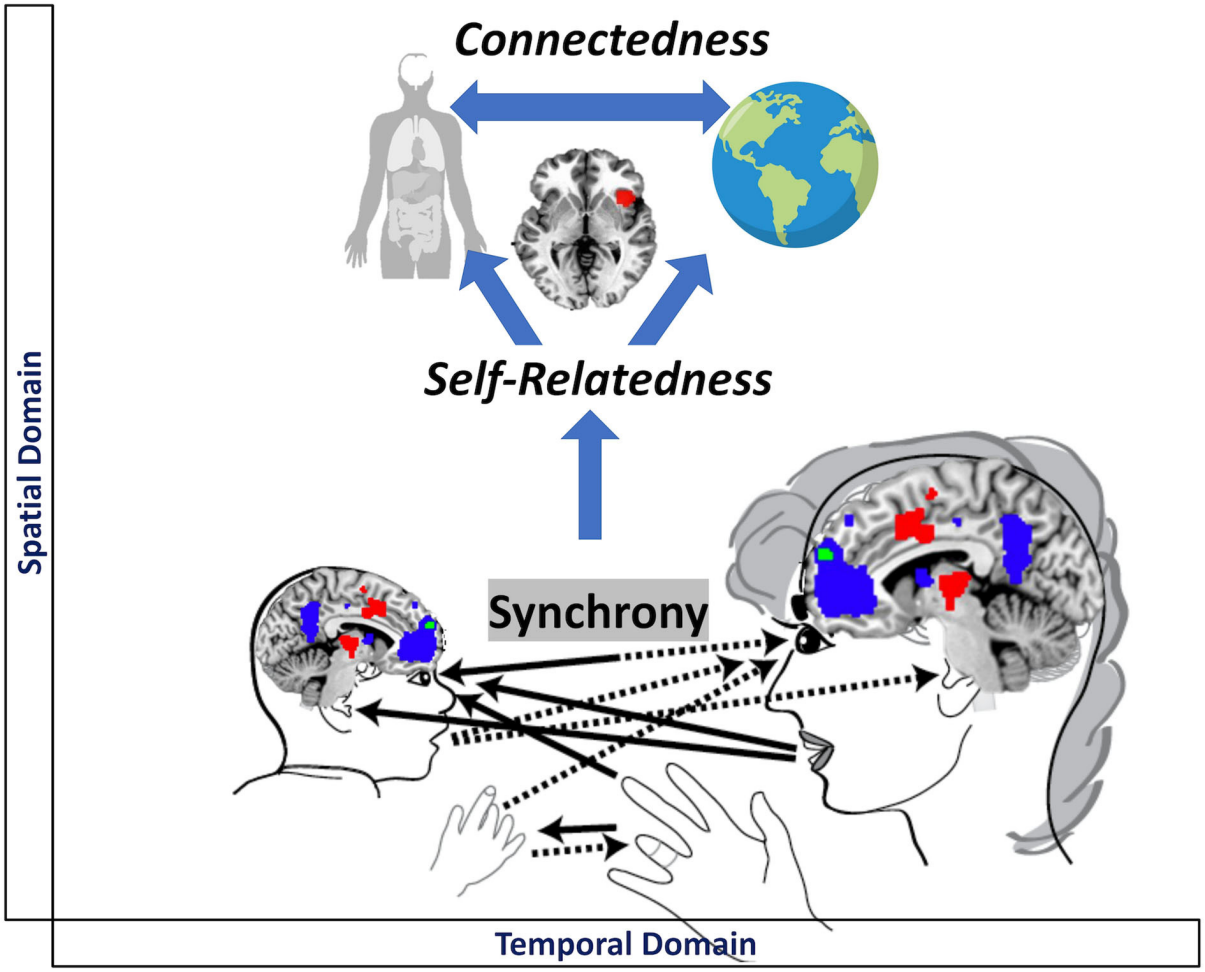
Visual representation of the mutual relationship between psychological and neuronal synchronization.

The construct of self-relatedness here seems to be very close to the larger construct of connectedness that has been used to describe the connection to the body (Porges, 2011), the self, to others, and the world in general (Watts et al., 2017). Congruently, in the field of psychedelic science, a group of scholars (Carhart-Harris et al., 2018) aim to develop an operational definition of connectedness that incorporates not just connectedness in the subjective sense, but also at its biological and behavioral levels. Recently in dynamic psychology and psychoanalysis, Mucci (2013, 2018a, 2022) proposes the construct of connectedness as fundamental in the social exchanges of human beings and in maintaining a bond to life and is itself a form of resilience against adverse life traumatic experiences that might disrupt our sense of self and relatedness with others (refer to Figure 2).

## The multilayer nested model of self

The attention of several authors has recently been elucidating the neural architecture related to the self. For example, Panksepp (1998) and Damasio (2010) associated a hierarchy of the self in the brain with bodily self, autobiographical self, and extended self.

Damasio (2010) in his model theorized the “proto self,” which generates primordial feelings and is largely subconscious. This bottom layer of self provides the grounding for the higher levels, such as the “core self,” which represents the transient relationship between an individual and the surrounding environment, and finally, the “autobiographical self,” which requires working and long-term memory.

A more radial-concentric approach to the brain is the three-layer anatomical model of the brain as proposed by Feinberg and Northoff (Northoff et al., 2011). These proposals parallel recent socio-psychological and phenomenological findings (Gallagher, 2000; Nelson et al., 2014) and support the evidence for a functionally constituted entity of the self ranging over multiple interacting levels from an unconscious, pre-reflective, and minimal self to a reflective phenotypic “idiographic” narrative self constituted by interpersonal and sociocultural experiences. These proposed hierarchies of self seem to be in line with Endel Tulving's three-layer theory of consciousness (Tulving, 2002): *anoetic* (unthinking forms of experience, which may be effectively intense without being “known”) *noetic* (thinking forms of consciousness, linked to exteroceptive perception and cognition) and *autonoetic* (abstracted forms of cognitions and perceptions and cognitions, which allow conscious “awareness” and reflection through episodic memories and fantasies. These layers, accordingly with Solms and Panksepp (2012), seem to be connected with some major evolutionary passages of the brain, such as (i) the evolution of the upper brainstem (up to the septal area) as related to *anoetic consciousness*, (ii) the evolution of lower subcortical ganglia and upper limbic cortical midline structures as related to *noetic consciousness*, and (iii) the evolution of higher neocortical functions and association cortices as related to *autonoetic consciousness*.

A recent evidence-based, large-scale fMRI meta-analysis in healthy subjects suggests a multilayered nested hierarchical model of self (Qin et al., 2020) including: (i) interoceptive self; (ii) extero-proprioceptive self; and (iii) mental self.

**Figure 3:**
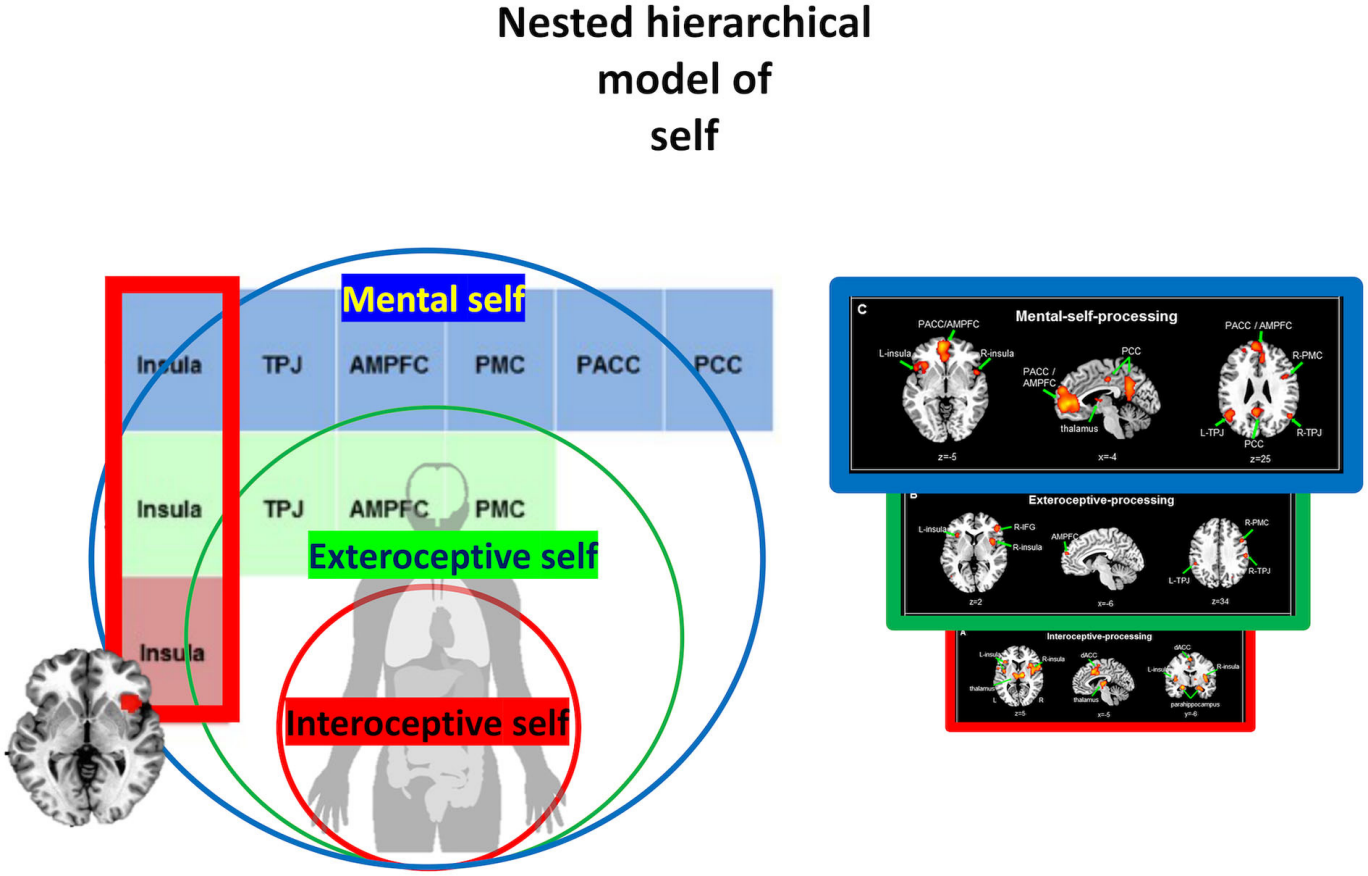
Visual representation of the nested hierarchical model of self. L, left; R, right; dACC, dorsal anterior cingulate cortex; IFG, inferior frontal gyrus; TPJ, temporo-parietal junction; AMPFC, anterior medial prefrontal cortex; PMC, premotor cortex; PACC, pregenual anterior cingulate cortex; PCC, posterior cingulate cortex.

The interoceptive self refers to the processing of the body's inner organs and was investigated through an fMRI task related to interoceptive awareness of one's own body. The extero-proprioceptive self focuses on external or proprioceptive bodily inputs and was investigated with fMRI studies focusing on external bodily-related inputs, such as facial or other proprioceptive inputs. Finally, the mental self was investigated considering all task employing trait adjectives or other self-related stimuli vs. non-self. Intriguingly, the studies related to the interoceptive self emphasizes the role of bilateral insula, dorsal anterior cingulate cortex, thalamus, and parahippocampus, which are also considered core regions of the salience network (Menon and Uddin, 2010). The extero-proprioceptive self yielded regions, such as the bilateral insula, interior frontal gyrus, premotor cortex, temporo-parietal junction (TPJ), and medial prefrontal cortex (MPFC). These regions share the processing of proprioceptive inputs related to the body, and this seems to be closely related to the concept of “embodied self” (Gallagher, 2005; Tsakiris, 2017). Finally, the mental self, related to fMRI studies, yielded DMN cortical midline regions, such as the medial prefrontal cortex and posterior cingulate cortex, as well as the regions included in the extero-proprioceptive self, most notably the bilateral TPJ, as well as regions of the interoceptive self, i.e., bilateral insula and thalamus. Together, these findings describe a hierarchical model of self (Qin et al., 2020) showing how regions of the interoceptive self are also included in the other layers (extero-proprioceptive and mental self) where they were complemented by additional regions extending the topography of the self. Moreover, this study also highlights the function of the insula, which seems to represent the common denominator for each level of self processing (refer to Figure 3). This hypothesis linking the functional role of the insula and the different layers of the sense of self seems to be further confirmed by the studies on macaques (Critchley and Seth, 2012), studies on the role of the insula and emotional awareness (Gu et al., 2013) and by its relevance in severe psychopathologies, such as anorexia (Esposito et al., 2018) and anxiety disorders (Lucherini Angeletti et al., 2021).

This nested topographical hierarchical model of self might be associated with and add more information to the proposed neuropsychodynamic nested model of self (Scalabrini et al., 2018).

**Figure 4:**
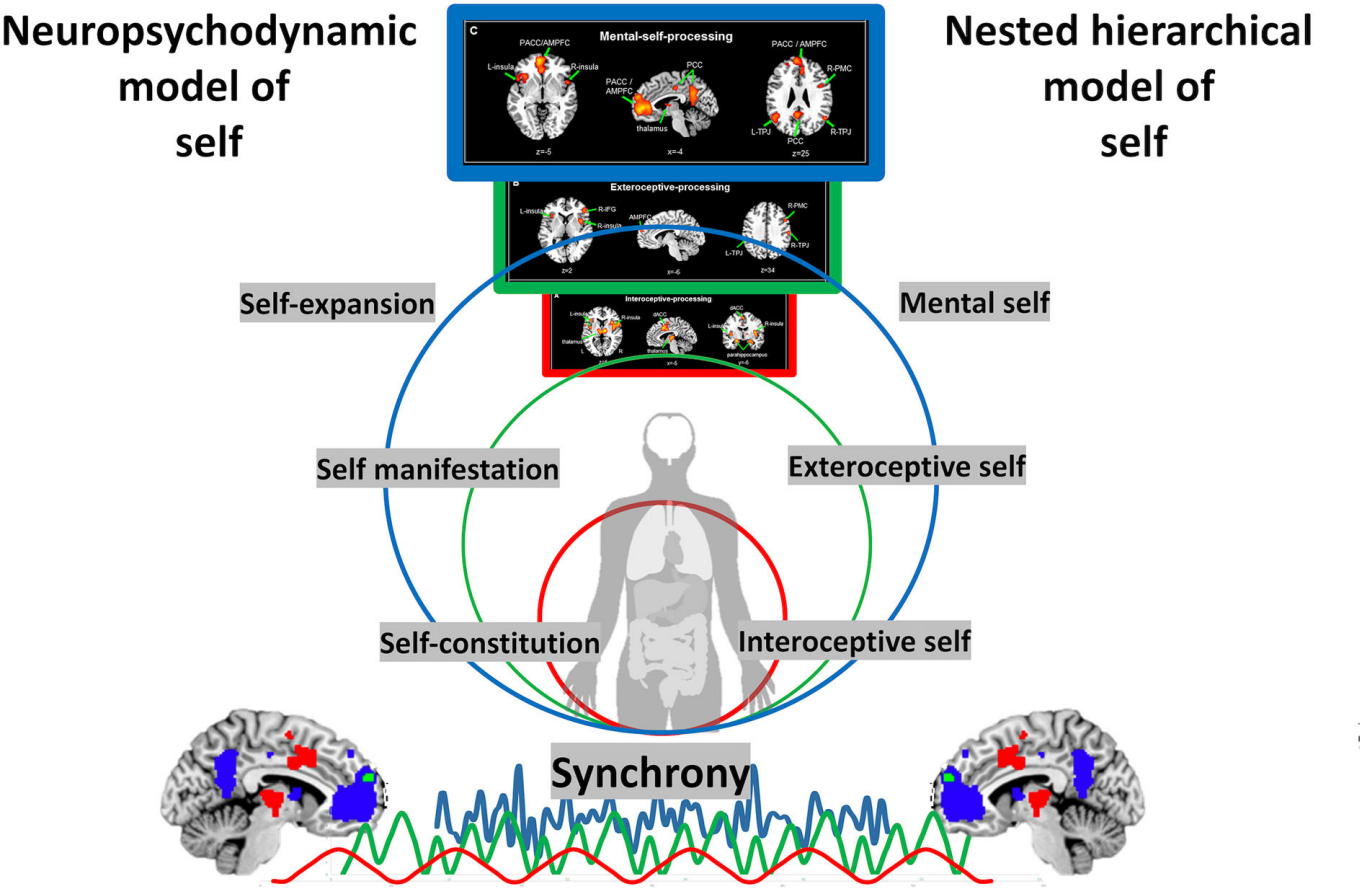
Conjunction of the neuropsychodynamic model of self and nested hierarchical model of self. Self-constitution, as related to the layer of interoceptive self, is linked with the ownership of one's own body, sense of agency and the capacity to distinguish the self from the non-self and the internal from the external (i.e., reality testing). Self-manifestation, as related to the layer of exteroceptive self, is particularly featured by the degree of integration of the self and significant others and by the actual experience and manifestation of the self with the external world. Finally, Self-expansion, as related to the layer or mental self, is characterized by the capacity to self-expand and bind the different information of various aspects of self and other into perception and memory.

In this context, the authors propose a multilayered model of the self-departing from the building blocks of relational alignment to the different layers of self, named: (i) self-constitution; (ii) self-manifestation; and (iii) self-expansion, which might somehow parallel the: (i) interoceptive; (ii) extero-proprioceptive; and (iii) mental self (refer to Figure 4)

As proposed by the authors (Scalabrini et al., 2018), relational alignment or synchronization is considered the prerequisite that gives the newborn the framework for the sense of subjectivity that is dependent on the first encounter with the other (e.g., the caregiver). The encounter with the other may facilitate (or not) the constitution and development of the self, depending on the degree of attunement that plays a fundamental role in shaping the sense of self, relatedness, and the capacity to regulate emotions and to mentalize among the complexity of psychological development (Schore, 2001a,b, 2012, 2021; Mucci, 2013, 2018a, 2021a,b).

Intriguingly, aging and dysfunctions have been related to decreased neuronal complexity in regions typically involved in “more cognitive” task (e.g., frontal and parietal lobes), while increased neuronal complexity has been found in regions, such as insula, limbic, and temporal lobe, typically considered as “emotional” and/or more specific to the self (Cieri et al., 2021). These findings further support the relevance of the self in development and aging of the individual.

As previously shown, this hypothesis is further supported by literature showing how the child's development and their growing brain are optimized where the provision of parental care is sensitively attuned to the infant's needs (Atzil and Barrett, 2017; Atzil et al., 2018 for a perspective). Atzil et al. (2018) propose how the insula and the regulation of interoception and allostatic needs of the child might play a fundamental role in the development of the self and of a social brain.

The emphasis on the interoceptive and allostatic needs of the child further supports the hierarchical model of self (Qin et al., 2020) and the importance of the insular cortex as a crossroads for the relation integration between internal and external stimuli (Craig, 2010; Menon and Uddin, 2010).

It seems that these synchronous relational aspects, together with the key role of the insula, are the prerequisite for the three layers of the self. Intriguingly, in a recent article investigating the spatiotemporal dynamics of the three layers of self in the spontaneous activity of the brain (Scalabrini et al., 2021b), the right anterior insula exhibited increasing centrality indices over the three hierarchical layers of self in comparison with all other regions implicated. High centrality and functional connectivity indicate that the right insula synchronizes and integrates the activity of the other regions of the self-networks. Functional connectivity allows for functional integration, that is, the degree to which a region pools or sums the activity of other regions within its own neural activity through synchronization (Deco et al., 2015). The increased high degree of centrality over networks enables the right insula to integrate interoceptive, exteroceptive, proprioceptive, and cognitive/mental functions linking the three layers of self-specific information which are thereby spatially or topographically nested within each other (Qin et al., 2020).

Moreover, the right insula exhibits longer time windows in its neural activity than both the left insula and the other regions of the different layers of self. Together, these findings suggest higher degrees of both functional and temporal integration in the neural activity of the right insula. This suits the right insula to ideally serve as a topographic and dynamic node or glue between the distinct layers of self and their high degrees of spatial nestedness and temporal continuity.

Taken together, here, we provide a parallel between these two models that give us a neuropsychodynamic topographical grounded model of self which carries major psychodynamic and neuroscientific implications.

**Figure 5:**
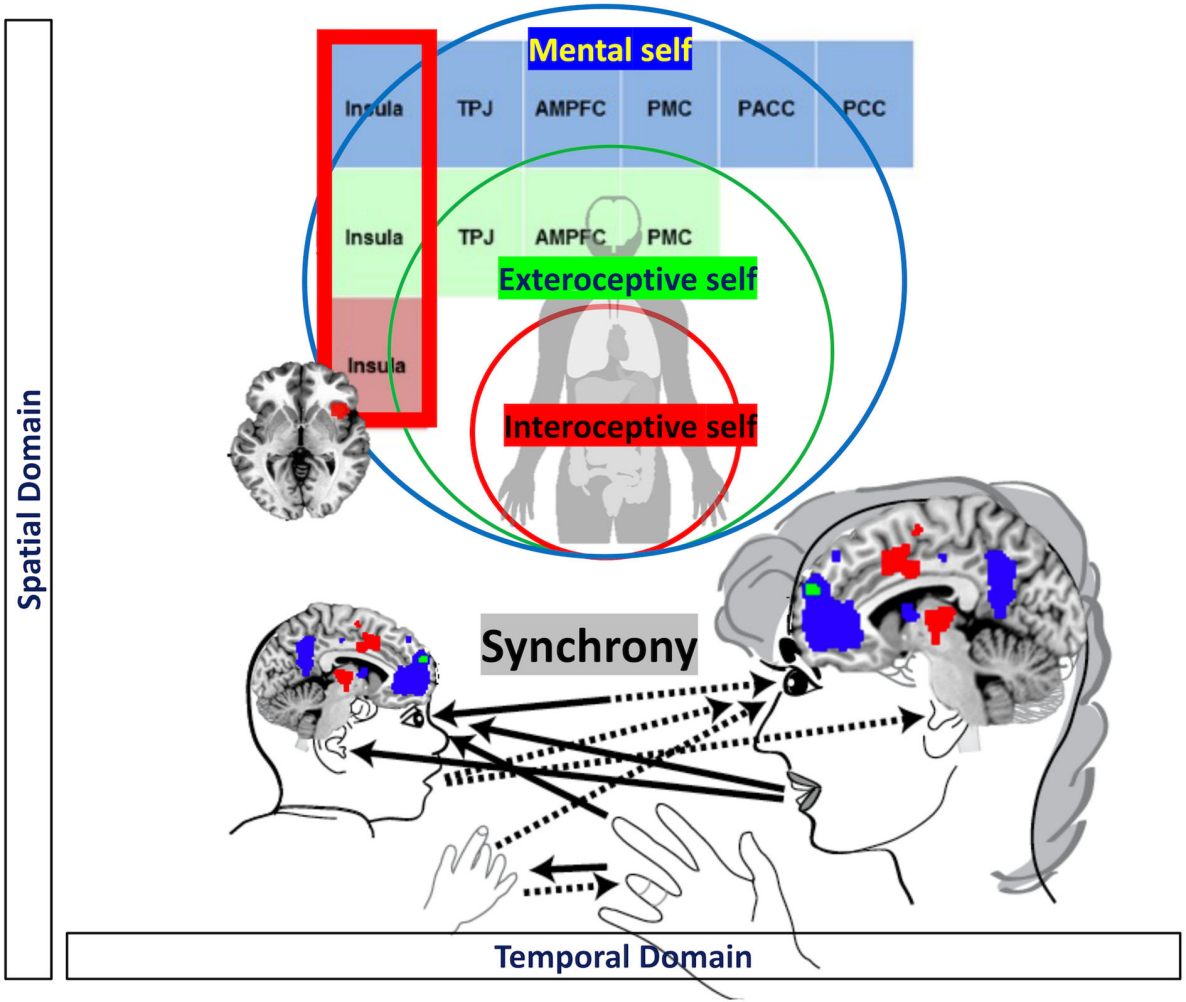
The role of anterior insula in biobehavioral synchronization between caregiver and child and in the nested hierarchical model of self.

We hypothesize that the right insula's key role in constituting spatial nestedness on the neuronal level, i.e., among the three layers of self-networks may also be in manifesting on the psychological level: self-specific interoceptive information may be contained and nested within the layer of self-specific proprioceptive and exteroceptive information which, in turn, may be nested and contained within the even more extended layer of self-specific mental or cognitive information (refer to Figure 5).

## From self to trauma – topographic-dynamic layers of trauma

We now assume that the nested hierarchy of self might be connected to the hierarchy of trauma, that is, the effects traumatic events can induce in the self and ultimately the brain. Our observations follow Clara Mucci's excellent account of trauma through interdisciplinary and clinical observation (Mucci, 2013, 2014, 2018a, 2019, 2021a,b, 2022): she distinguishes three layers of inter-personal trauma which we, in a second step, connect with the different layers of self in its nested neural hierarchy.

Mucci (2013, 2018a, 2022) distinguishes between three levels of inter-personal trauma as distinct forms and different degrees in the severity of disrupted attachment across the life span from infancy to adulthood. Notably, all three levels are not separated entities but rather refer to different degrees of an underlying continuum of trauma severity and depth. Hence, the distinction of the three levels is more heuristic, serving conceptualization; they should not be confused with the actual reality itself where one can observe multiple transitions and overlaps with possible cumulative effects and influences between one level and the other.

### First layer of trauma I – early relational trauma, subcortical regions, and interoceptive self

The first, most basic level of inter-personal trauma concerns what Mucci (2013, 2018a), and Schore (2003a,b, 2009) describe as “early relational trauma.” This primarily goes back to early infancy, where the infant suffers from a lack of attunement and synchronization with the caregiver, who is unable to provide secure, stable, and continuous care and containment for the infant (Schore, 2003a,b). This creates disorganized attachment (Liotti, 1992) in the infant and makes it prone to most basic dissociative responses (Schimmenti and Caretti, 2016; Farina et al., 2019; Scalabrini et al., 2020a; Mucci, 2021a,b; Mucci and Scalabrini, 2021) in childhood and later in adulthood. Moreover, the infant or child suffers from affective dysregulation with hypoarousal, which later may translate into high vulnerability for depression, anxiety, and the development of trauma-related personality disorders.

Neuronally, the early relational trauma has been associated with various cortical and subcortical brain regions that belong to the limbic system, including the insula, the amygdala, and the orbitofrontal cortex (Siegel, 1999; Schore, 2003a,b). Interestingly, these regions strongly overlap with those recruited during the most basic layer of self in the nested neural hierarchy, the interoceptive self: subcortical regions such as the thalamus and those in midbrain and even brain stem, as well as cortical regions such as the insula mediate this most basic layer of the interoceptive self.

These regions predominantly process and integrate interoceptive stimuli and inputs from the subject's own inner body and, in later stages, their integration with exteroceptive inputs. This is the most fundamental layer of the self which ties it closely to the body and environment, i.e., the most basic intero- and exteroceptive inputs, any infant (and later child and adult) receives. Disturbances in these regions' interoceptive processing consequently lead to major disturbances in the most basic sense of self, i.e., the interoceptive self.

### First layer of trauma II – from irregular/absent interoceptive input to disordered attachment

How is this most basic and fundamental layer of the interoceptive self related to the symptoms of early relational trauma? If the caregiver like the mother does not provide proper basic and affective care, such as nutrition and emotional mirroring, in a regular and predictable way, the interoceptive inputs to the subcortical regions and the insula are highly irregular and/or absent. What is described as “*disorganized attachment*” on the psychological level (Liotti, 1992; Schore, 2003a,b, 2009; Mucci, 2013, 2018a, 2022) may then be related to “disorganized interoceptive input processing” on the level of the brain (refer to Atzil et al., 2018): irregular or even absent interoceptive inputs will make it rather difficult for these limbic subcortical and cortical regions to develop a proper dynamic, that is, in neuronal spatiotemporal terms a power spectrum with the balance of slow and fast frequencies in a scale-free and temporally well integrated and nested way.

Specifically, there may be a disbalance of slow and fast frequencies which, in turn, impedes all subsequent processing in these limbic subcortical nuclei and related cortical regions, including their integration of the various interoceptive (and later also exteroceptive) inputs. If, however, intero-intero/exteroceptive integration is impaired, the sense of their interoceptive self may become unstable and fragile – the self of those subjects shows a lack of energy with hypoarousal (as eventually related to the Nucleus Basalis Meynert), affective dysregulation (as eventually related to serotoninergic and adrenergic/noradrenergic subcortical regions; refer to Panksepp and Biven, 2012), and dissociation proneness (as related to the lack of intero-and exteroceptive integration and decreased connectivity; Scalabrini et al., 2020a).

**Figure 6:**
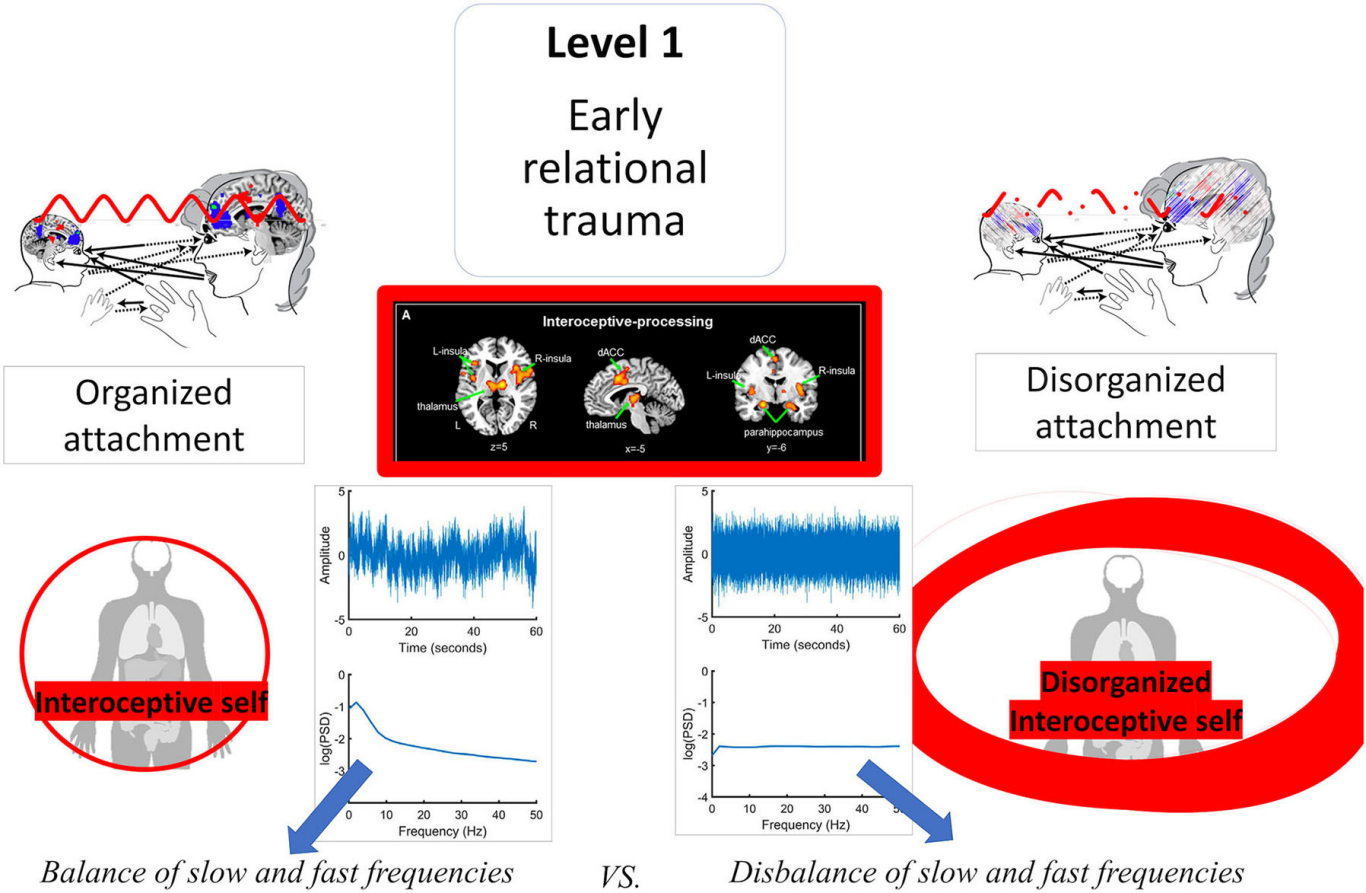
Visual representation di *disorganized interoceptive self*.

Together, irregular or absent interoceptive inputs through the caregiver may translate into corresponding neuronal topographic disbalances and dynamic irregularities in the power spectrum predominantly in limbic subcortical-cortical regions. The subcortical-cortical regions' abnormally slow-fast balanced power spectrum with decreased temporal nestedness, in turn, may be related to decreased arousal, affective dysregulation, and an unstable or fragile interoceptive self. These processes manifest in what has been described as “*disorganized attachment*” on the psychological level (Liotti, 1992; Mucci, 2018a) that neuronally may be reflected in the brain's “*disorganized topography and dynamic*” as the basis for the so-called psychological baseline and social brain (Northoff, 2016; Atzil et al., 2018; Scalabrini et al., 2022; refer to Figure 6).

### Second layer of trauma I – from maltreatment and abuse to the fragmentation of self

The second level of inter-personal trauma concerns what Mucci (2018a) describes as “maltreatment and abuse.” This may concern events that often exceed beyond the “normal” ranges of human experience like maltreatment, severe emotional and physical deprivation, incest, and abuse. These events can occur at every time of the life cycle, including in infancy or early childhood where they may exert major reverberation for the psyche.

As focused here, these events may mainly occur in infancy and early childhood where they leave major consequences for later adulthood. Dissociation and/or identification with the aggressor characterizes this second level of trauma described by Mucci (2013, 2018a, 2022) which adds on to the first level, the non-intentional disattunement of the caregiver and places additional stress and dynamic consequences of severe abuse or maltreatment and severe deprivation (and the identification with the aggressor, with the internalization of guilt and rage, often revolved around the self).

In the research, maltreatment and abuse have been indicated as a vulnerability to develop disorganized attachment in childhood and possible dissociative behaviors (Main and Hesse, 1990; Liotti, 1992; Carlson and Sroufe, 1995; Draijer and Langeland, 1999; Ainsworth and Eichberg, 2006). In addition, it creates a dissociated personality and internalized victim/persecutor dyad (refer to Kernberg, 1967; Fonagy and Bateman, 2007; Mucci, 2018a) where one identifies with the victim and the related effects (low self-esteem, blame, shame, guilt), yet also identifies with as an internal persecutor position of which the subject is not aware, and identifies with the effects of the persecutor which include violence, hate, and aggressiveness (often revolving around one's body). The identification with the aggressor (Ferenczi, 1932a/1988,b) is a way that the personality adopts to survive. The internalized persecutory parts can act against oneself (damaging oneself and one's resources) or against the other (becoming actually violent against another individual).

The second layer of the nested hierarchy of self therefore extends beyond the interoceptive self to its proprio-exteroceptive input from the outside of the own body and others' relative to the own body.

**Figure 7:**
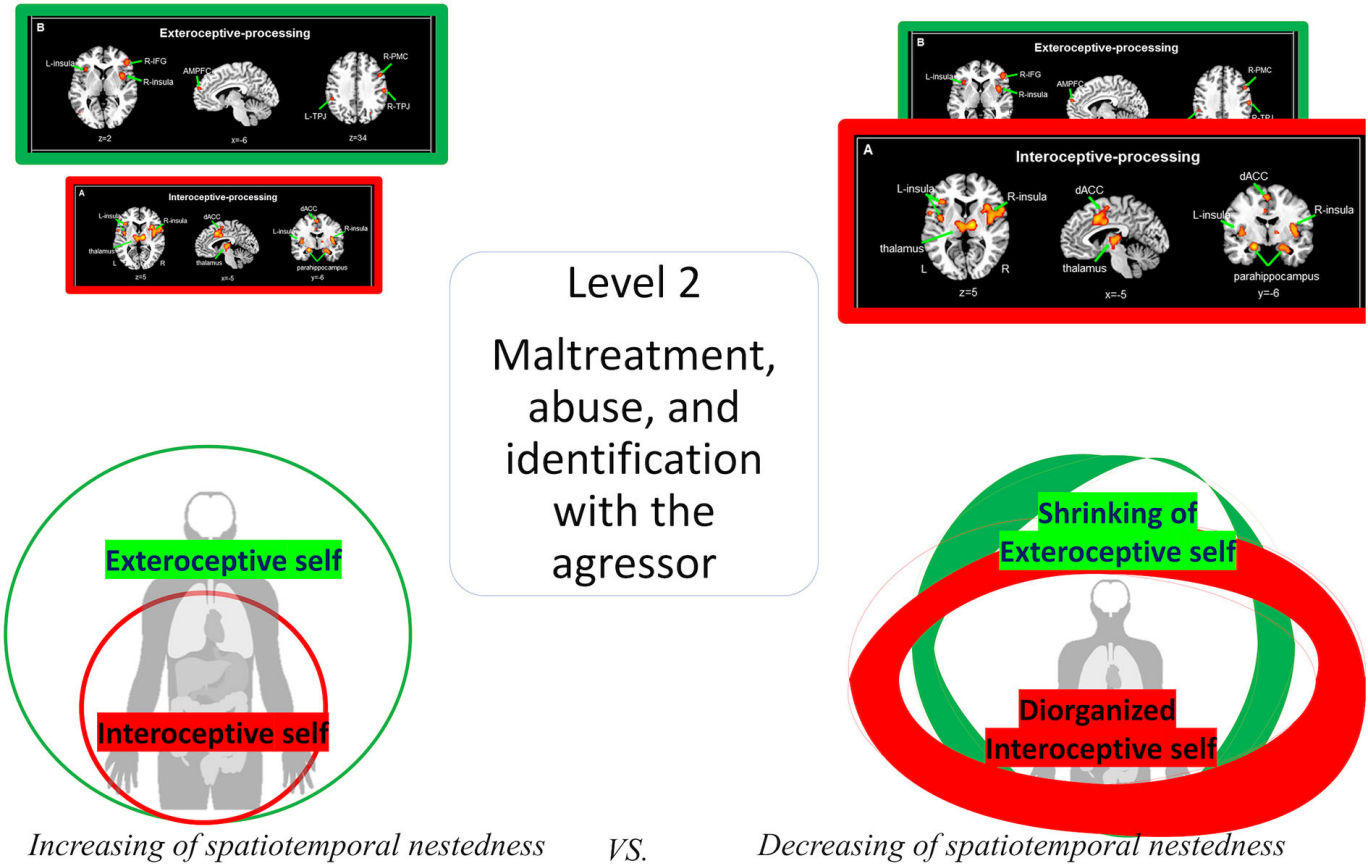
Visual representation of the shrinking of *proprio-exteroceptive self*.

This is neuronally reflected in the recruitment of regions, such as the anterior medial prefrontal cortex, premotor, fusiform face area, and temporo-parietal junction, that typically process these kinds of inputs (refer to above and Qin et al., 2020). Importantly, this proprio-exteroceptive layer of self builds and nests upon the more fundamental first layer, the interoceptive input layer of self: the latter and its regions are integrated within the former. Such nested hierarchical relationship carries major implications for how the self can cope with trauma (refer to Figure 7).

### Second layer of trauma II – topographic-dynamic re-organization of brain and self

One may now postulate that the second level of trauma with abuse, maltreatment, and/or physical and emotional deprivation may primarily affect the extero-proprioceptive layer of the self by disrupting the activity of its respective regions. If that extero-proprioceptive input layer is disrupted, neural activity will “fall back” onto the more basic and fundamental layer, the interoceptive input layer.

At the same time, the extero-proprioceptive layer of self may be split off from the nested hierarchy, including its containment or nesting within the interoceptive layer of self. In a way similar to Russian dolls, such that this is like “taking out” one of the more intermediate-sized Russian dolls which then, relatively speaking, will enlarge the next larger one in a disproportionate way. Though, one needs to be more careful in this interpretation. The traumatized layer is not completely “taken out” as it is still present as described by Ferenczi and still manifests in the various affective-emotional symptoms related to the trauma. In this case, the trauma leaves its traces at a bodily interoceptive level. In contrast, the extero-proprioceptive level, instead of being literally “taken out,” “shrinks” in an abnormal way whereby it may more or less disappear into the previous one, the interoceptive layer.”

Psychologically, this means that the extero-proprioceptive self may become abnormally small, shrink, and no longer be visible within the more overarching interoceptive layer of self. Put in spatiotemporal terms, this means that the spatial extension and the temporal range of the extero-proprioceptive self may be reduced to the ultimately smaller spatial and temporal ranges of the interoceptive self. The nested hierarchy of the self and brain thus may be disrupted in its degree of temporal and spatial nestedness between the different layers. The extero-proprioceptive self may no longer be perceived as related to the self, thus creating the circumstances for aggressive behaviors manifested toward the bodily self or toward the external world.

Trauma may then be understood as disruption of the temporo-spatially nested hierarchy of both the brain and self.

How can the person defend its own self and react to its decreased temporo-spatial nestedness of its different layers of self? How is such “filling of the extero-proprioceptive missing” manifested on the psychological level?

As theorized by Mucci (2013, 2018a), we can see the victim/persecutor split in severe personality disorders, which are especially common in young adults nowadays.

On the one hand, we can observe how borderline patients self-cut or try to commit suicide attacking their own body subjugated by the disorganized interoceptive self. Contrastingly, severe narcissists rely on the mental effort to maintain a sense of pathological grandious self which damages their own resources (e.g., time and intelligence) to protect their own inner fragility and low self-esteem (for an fMRI study on narcissistic grandiosity refer to Scalabrini et al., 2017). The identification with the aggressor, with the internalization of the rage against one's body or against the other, explains much of the destructiveness against self and others in personality disorders and other severe mental pathology (Mucci, 2013, 2017, 2018a).

In sum, the changes related to the second layer of trauma reflect the re-organization of (i) the spatial topography of the nested neural hierarchy of self and (ii) its related power spectrum with its slow-fast balance. Hence, what we, from the outside, describe as disruption of the temporo-spatial nestedness of the neural hierarchy of self may, from the subject's inside or its first-person perspective, be a topographic-dynamic re-organization of its own self to cope with the trauma albeit being ultimately maladaptive.

### Third layer of trauma – from massive trauma to topographic-dynamic re-organization of the mental or cognitive self

There are events, such as genocide, war, and rape/abuse, that lie outside the “normal” range of human experiences. How does the brain and self react to such cumulative massive traumatic experiences?

This depends first on the intrinsic factor like the stability of the nested hierarchy of the self and second on the severity of traumatic occurrence and its cumulative effects. The same trauma may have different impacts depending on whether it occurs during infancy, adolescence, or adulthood as these are related to the different layers of the nested hierarchy of self. It also depends upon the severity of trauma and the history of cumulative trauma experiences by the subject: the more severe a trauma and the more severe the history of traumatic experiences in the past (1st and 2nd levels), the deeper it will reverberate into the deeper layers of self disrupting its own stability.

One may assume that massive traumata primarily affect the most upper layer of self, the mental or cognitive self, and its underlying regions such as the cortical midline structures. Neural activity in these regions may be diminished and reduced in the face of such severe trauma which is indeed supported by various results (Mucci, 2018a, 2021a,b; Scalabrini et al., 2020a; Mucci and Scalabrini, 2021). The self as mental baseline or default mode of the brain (Northoff, 2016; Scalabrini et al., 2018, 2021a) is put “out of order.” The mental self as the most upper layer of self is consequently diminished.

This, in turn, changes the whole nested hierarchy of self. The most upper layer, the mental self, is now abnormally “shrinked” which, relatively speaking, renders the interoceptive and extero-proprioceptive layers of self abnormally strong. This is, for instance, reflected in the often observed increase of anxiety with extreme interoceptive awareness in these subjects that often suffer from various psychopathological syndromes, such as depression, anxiety, trauma-related, and personality disorders (Mucci, 2018a; Scalabrini et al., 2018, 2021a).

**Figure 8:**
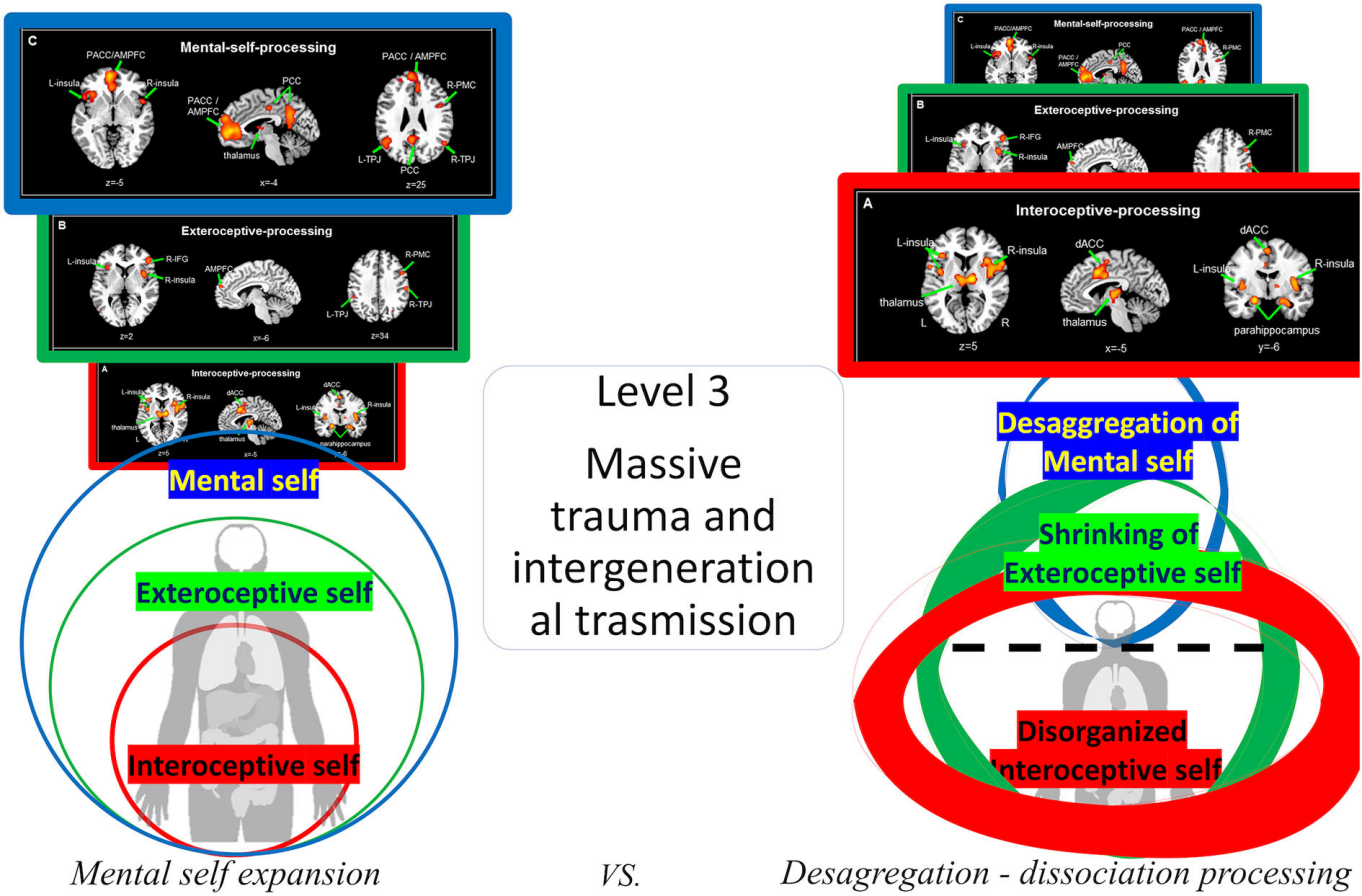
Visual representation of the *desaggregation of the mental self*.

At the same time, the mental or cognitive features of the self may be “split” from its lower nesting layers, the intero- and extero-proprioceptive layers of self. That may be reflected in the various compartmentalization and detachment symptoms of dissociation (Scalabrini et al., 2020a). Dissociation thus may be understood as the disruption of the “glue” or nestedness among the different layers of self – this, as we postulate, may be traced to a corresponding disruption in the nestedness of the neural layers of self (see Figure 8).

### Dissociation as a disruption of temporo-spatial nestedness

Dissociation plays a key role in trauma which, as we assume, connect at the neural and mental levels. What is described as dissociation of parts of the self on the psychological level may be mediated by a corresponding dissociation on the neural level of self: the neural level of the default-mode network mediating the mental or cognitive layer of self dissociates from its nesting neural layers, the subcortical-cortical layers of both interoceptive and extero-proprioceptive self. Correspondingly, the dynamics of the power spectrum will dissociate in some of its parts, such as its slower and/or faster frequency ranges. This results in the disruption of the temporo-spatial nestedness of the brain's hierarchical topography and dynamics. As in the case of the second layer of trauma, subjects may react to massive trauma by topographic-dynamic re-organization of their brain and its self by strengthening the respectively preserved layers.

What happens when the subjective synchronous experience with the self, the body and the world is disrupted?

In a recent article by Scalabrini et al. (2020a), the central role of the right anterior insula is considered as the central hub for the temporo-spatial synchronization between body and brain, that is disturbed in dissociation. Such temporo-spatial desynchronization of the body–brain connection should disrupt the first-person perspective (Tallon-Baudry et al., 2018): instead of being perceived in relation to the own person (in a first-perspectival mode), the contents would remain detached from the self and its bodily-based first-person perspective: the contents will then be perceived as such but in such a way that they remain unrelated to the person and its bodily-based first-person perspective – the contents will consequently be perceived as foreign or alien, e.g., as non-self-related, thus reflecting what, on the symptomatic level, is described as detachment symptoms.

**Figure 9:**
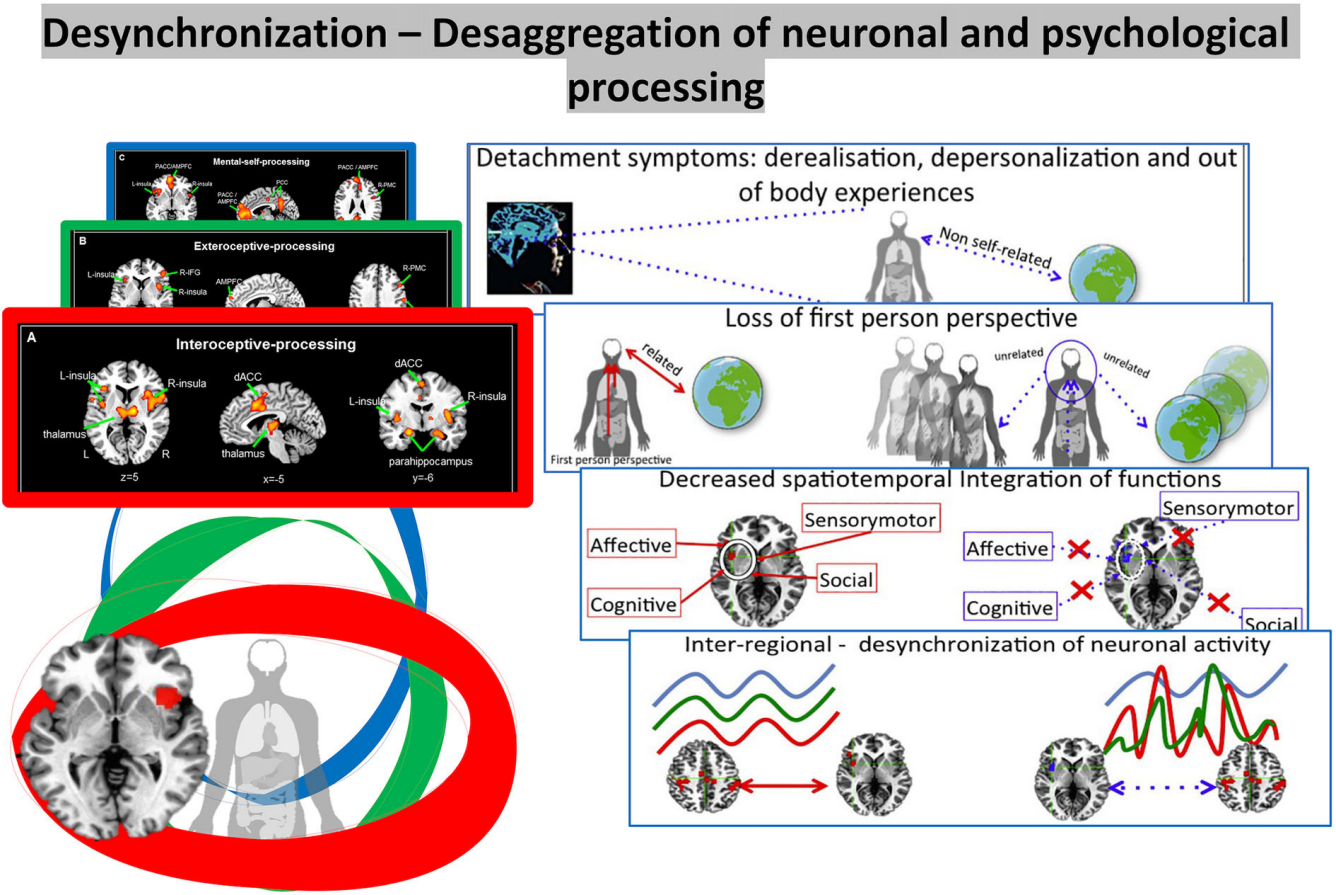
Visual representation of dissociation and traumatic re-organization of nested hierarchy of self.

More specifically, the desaggreation of intero-exteroceptive function based on the impaired spatiotemporal integration leads to the alterations in embodiment and, even further, the disruption of our self's relationship with the others and the environmental context. This failure of integration can thus be seen as a disconnection that induces an instant collapse of both subjectivity and intersubjectivity, supporting affective dysregulation (Schore, 2001a,b, 2003a,b; Mucci, 2018a; Schimmenti and Sar, 2019; Scalabrini et al., 2020a,b; Cavicchioli et al., 2021). Such disruptions of embodiment and first-person perspective are well reflected in trauma-related disorders. Stressful stimuli, especially those associated with painful emotional effects, are thus not experienced in consciousness, and they are associated with what Bromberg (2014) terms “not-me” self-states. At the same time, intero-exteroceptive desaggregation leads to disruption of the self's attunement with the other and consequently the world. This reinforces a vicious circle of traumatic experience that, through dissociation, are intergenerationally transmitted between caregivers and child (Mucci, 2013, 2018a, 2022) (see Figure 9).

## Conclusion: Synchrony between and beyond layers – topographical and dynamic re-organization of the nested hierarchy of self and its biobehavioral-affective regulation

**Figure 10:**
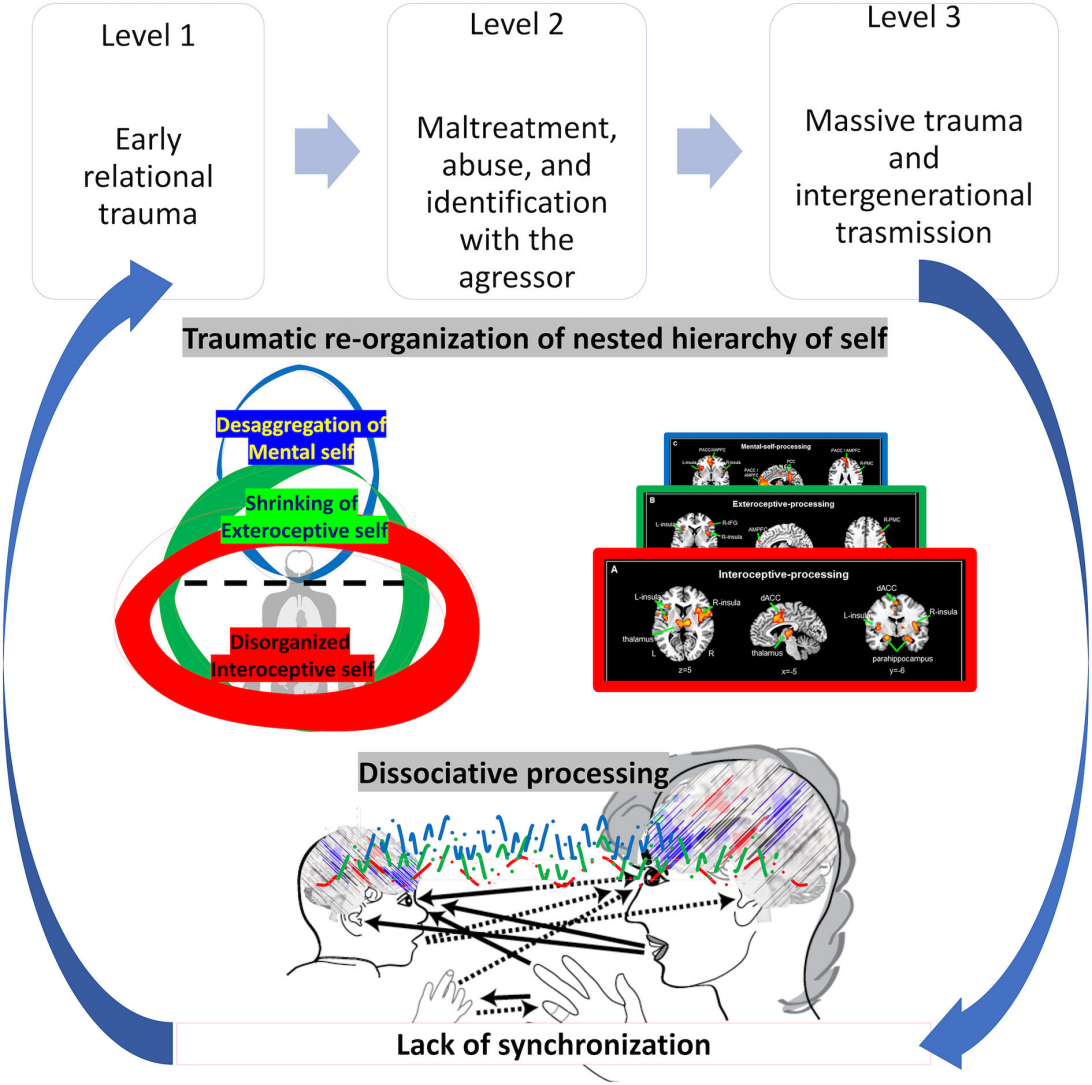
Visual representation of the traumatic dynamic and topographical re-organization of nested hierarchy of self.

We characterize the effects of trauma by an abnormal shift in the topography and dynamic of the nested hierarchy of self. The trauma-related symptoms are conceived here as topographic-dynamic re-organization of self and brain albeit in a maladaptive way, that we call the traumatic re-organization of the nested hierarchy of the self (see Figure 10 for a summary).

As we recently proposed (Northoff and Scalabrini, 2021), the aim of a neuropsychodynamic informed psychotherapeutic process is to (i) to reverse such maladaptive topographic-dynamic reorganization of brain and (ii) to establish a more adaptive and stable temporo-spatial nestedness of brain and self thereby re-establishing a proper nested hierarchy of self. This process might serve to re-establish the subjective sense of integrity, coherence, and continuity of self over time and space, “*the capacity to feel like one self while being many*” (Bromberg, 1996, p. 1. 166)

Moreover, to re-establish and integrate the sense of self of the patients, the therapist should act as a regulator of different affective and self states working at the edges of affective dysregulation and in-between different states of mind (Mucci, 2013, 2018a, 2021a,b; Mucci and Scalabrini, 2021). As Schore writes: “*with early forming severe right-brain pathologies, the clinician's primary function is as an affect regulator for the patient's primitive, traumatic states, including those affective states that are walled off by dissociation*” (Schore, 2003a,b p. 246).

Dissociation, as we suggested before, operates in terms of lack of integration and synchronization between the different layers of the self (Scalabrini et al., 2020a). As a consequence, moving beyond dissociation means to re-establish the nested hierarchy of the self system to process at the mental level (mental self) the perception of external stimuli (proprio-exteroceptive self, i.e., information coming from the relational environment) and integrate them with internal stimuli (interoceptive self, i.e. information from the body, the “felt experience” - Craig, 2010, 2011).

Our claim seems to perfectly fit to what Mucci (2021a,b) and Schore (2011) describe in terms of dissociative processing:

*dissociation thus reflects the inability of the right brain cortical sub-cortical implicit self system to recognize and process the perception of external stimuli (exteroceptive information coming from the relational environment) and on a moment-to-moment basis integrate them with internal stimuli (interoceptive information from the body, somatic markers, the ‘felt experience'). This failure of integration of the higher right hemisphere with the lower right brain system and disconnection of the central nervous system from the autonomic system induces an instant collapse of both subjectivity and intersubjectivity. Stressful effects, especially those associated with emotional pain, are thus not experienced in consciousness, (Bromberg ‘not-me' self-states)*. (Schore, 2011, p. xxxiii)

Consequently, healing the self and re-establishing the sense of self-continuity beyond the dissociation of its trauma may primarily require one to re-establish or re-organize the topography and dynamic of the nested hierarchy of self and its brain through spatial and synchronic means. Synchronization here refers to what Feldman terms as “social synchrony” in mother-child dyads: “*a move from a focus on one-brain functioning to understanding how two brains dynamically coordinate during real-life social interactions*” (Levy et al., 2017, p. 1036). This is similar to what we defined as “relational alignment” (Scalabrini et al., 2018) and Schore (2021) described as “*interbrain synchrony*” referring to an alignment of brains between two individuals, that is the “*simultaneous changes of emotional energy within the brains of both members of the dyad. This mutual entrainment of brain during moments of synchronization triggers an amplified energy flow, which allows for a coherence or organization of self and its implicit and explicit conscious experience.”* (Schore, 2021).

At a psychotherapeutic level, this seems to resemble to what has been theorized as “*critical or now moments in psychotherapy”* by Stern (2004) and the Boston Change Process Study Group (2005, 2013, 2018). For the BCPSG, the goal in psychotherapy is to share similar mental landscapes to understand and be understood for the re-organization of the sense of self and relatedness. This intersubjective sharing includes both the explicit verbal meaning of what one says and the implicit nonverbal meaning and in any event in time, “*intersubjective sharing*” is the primary goal of the therapy. The units of interaction are called relational moves and the goal of relational moves is to adjust or regulate the “*intersubjective field*,” that is, the shared mental/feeling landscape. These moves can consist of a spoken phrase, a silence, a gesture, or shift in posture, or a facial expression. Moving along can lead to sudden dramatic therapeutic changes by way of “*now moments*” and “*moments of meeting*.” That in our terms might be considered as moments of desynchronization and moments of synchronization. These moments shape the intersubjective field that gets suddenly reorganized: this occurs when the current state of implicit relational knowledge is sharply thrown into question and basic implicit assumptions about the relationship are now put in the discussion.

There is a mounting affective charge, which can be more or less regulated. The situation emerges unexpectedly and something must be done (including the option of doing nothing). Ideally following these critical moments “moments of meeting” or new moment of synchrony might take place to resolve the crisis. The moment of meeting seeks to use the disorganization of the now moment to enlarge the intersubjective field in ways not thought of before to reconnect the different layers of self. The therapist needs to synchronize (virtually or symbolically) her/his larger (spatial-topographic and temporal-dynamic) scales of her/his nested hierarchy of self to the more restricted of his client's traumatized self. These moments of meeting or synchrony require an authentic response finely matched to the momentary local situation to provide the client with the opportunity to integrate and nest her/his own more restricted spatiotemporal scales of her/his interoceptive self in a virtual, i.e., inter-personal way into the larger ones of her/his therapist.

A moment of meeting or synchrony requires an authentic response finely matched to the momentary local situation. It must be spontaneous and carry the therapist's personal signature. In that way, it reaches beyond a technical, neutral response and becomes a specific fit to a specific situation. These moments have been lived through together. That, in turn, will create the basis of a “psycho-physiological secure baseline” (refer to Northoff, 2016; Northoff et al., 2022; Scalabrini et al., 2022) to maintain a continuous and temporally extended and integrated sense of self and sense of relatedness that will allow the client to process the traumatic input relationships in a non-threatening and non-disrupting way for her/his own self way without becoming fragmented or dissociated. This result in a re-organization of the border between order and chaos, of coherence and complexity. The therapist and the patient have created an expanded intersubjective field that opens up new possibilities of ways of being with one another where the traumatic input relationships associated with the own interoceptive self are now integrated and nested virtually (or symbolically) within the therapists' larger spatiotemporal scales.

Practically speaking, the use of synchronic now moments in psychotherapy involves spontaneity and authenticity. Here, we propose to use temporo-spatial coordinates in the psychotherapeutic setting to work within the transference-countertransference matrix of the therapist–patient dyad. Working through synchrony, space, self-relatedness, and affective regulation might provide a more comprehensive, basic, and extensive operating field that also embeds and contains affective, social, cognitive functions within a larger more comprehensive context. This process aims to shape the temporal dynamic flow of the patient's neural and psychic activities to re-organize the traumatized self and consequently change the patient's baseline organization of the self and its spontaneous psychic and neuronal activities (Northoff and Panksepp, 2008; Scalabrini et al., 2018, 2022; Mucci, 2021a,b; Northoff et al., 2022).

## Data availability statement

The original contributions presented in the study are included in the article/supplementary material, further inquiries can be directed to the corresponding author.

## Author contributions

All authors listed have made a substantial, direct, and intellectual contribution to the work and approved it for publication.

## Funding

This project/research was supported by “Search for Excellence – UdA” (University G. d'Annunzio of Chieti-Pescara) to AS for the project SYNC (The Self and its psYchological and Neuronal Correlates – Implications for the understanding and treatment of depression as a disorder of Self) and by the Michael Smith Foundation Canada Research Chair, by the grant from the Ministry of Science and Technology of China, National Key R&D Program of China (2016YFC1306700) and from the European Union's Horizon 2020 Framework Program for Research and Innovation under the Specific Grant Agreement No. 785907 (Human Brain Project SGA2), the ERANET grant, NFRF grant, and Team grant from uOMBRI to GN.

## Conflict of interest

The authors declare that the research was conducted in the absence of any commercial or financial relationships that could be construed as a potential conflict of interest.

## Publisher's note

All claims expressed in this article are solely those of the authors and do not necessarily represent those of their affiliated organizations, or those of the publisher, the editors and the reviewers. Any product that may be evaluated in this article, or claim that may be made by its manufacturer, is not guaranteed or endorsed by the publisher.
